# The association between depressive symptoms and medication adherence among polypharmacy older adults

**DOI:** 10.1192/j.eurpsy.2024.748

**Published:** 2024-08-27

**Authors:** G. Alhashem, R. Alyasery, S. Al-Hassan

**Affiliations:** ^1^Pharmacy, AlSafwa University College, Karbala, Iraq

## Abstract

**Introduction:**

Among many polypharmacy term definitions, the most common definition refers to the concurrent use of five or more medications. Multiple medication administration is highly prevalent in older populations with multimorbidity. Apart from polypharmacy impacts on physical health, it might be detrimental to mental health.

**Objectives:**

The present study aims to evaluate the association between depression and poor adherence in multimorbidity Iraqi older population using five or more medications.

**Methods:**

This cross-sectional study was conducted in Iraq during July and August 2023, involving a sample of 196 older adults recruited from private clinics and hospital clinical medicine wards, all of whom had polypharmacy regimens. The questionnaire includes age, gender, medication regimen adherence and Patient Health Questionnaire-8 (PHQ-8) using a cutoff score of 10. Chi-square and binary logistic regression were performed to determine the association between poor adherence and the presence of depressive symptoms.

**Results:**

A total of 196 respondents, mean age = (61±11.4), 49 (25%) male and 147 (75%) female, 178 (90.8%) good adherence and 18 (9.2%) poor compliance, 81 (41.3%) participants have PHQ-8 score was equal or less than ten while 115 (58.7%) have PHQ-8 score was more than 10. Depressive symptoms and patient adherence showed a significant association (*p* = 0.02). Moreover, poor adherence polypharmacy participants were more likely to have depression odd ratio (OR) = 3.9, 95% confidence interval (CI = 1.09 – 13.9; *p* = 0.036).

**Conclusions:**

Our findings suggest that depressive symptoms are associated with poor adherence polypharmacy older adults and, highlighting the importance of addressing medication management and mental health in this population.

**Disclosure of Interest:**

None Declared

